# Control of Cross Talk between Angiogenesis and Inflammation by Mesenchymal Stem Cells for the Treatment of Ocular Surface Diseases

**DOI:** 10.1155/2016/7961816

**Published:** 2016-03-24

**Authors:** Fei Li, Shao-zhen Zhao

**Affiliations:** Tianjin Medical University Eye Hospital, The College of Optometry, Tianjin 300384, China

## Abstract

Angiogenesis is beneficial in the treatment of ischemic heart disease and peripheral artery disease. However, it facilitates inflammatory cell filtration and inflammation cascade that disrupt the immune and angiogenesis privilege of the avascular cornea, resulting in ocular surface diseases and even vision loss. Although great progress has been achieved, healing of severe ocular surface injury and immunosuppression of corneal transplantation are the most difficult and challenging step in the treatment of ocular surface disorders. Mesenchymal stem cells (MSCs), derived from various adult tissues, are able to differentiate into different cell types such as endothelial cells and fat cells. Although it is still under debate whether MSCs could give rise to functional corneal cells, recent results from different study groups showed that MSCs could improve corneal disease recovery through suppression of inflammation and modulation of immune cells. Thus, MSCs could become a promising tool for ocular surface disorders. In this review, we discussed how angiogenesis and inflammation are orchestrated in the pathogenesis of ocular surface disease. We overviewed and updated the knowledge of MSCs and then summarized the therapeutic potential of MSCs via control of angiogenesis, inflammation, and immune response in the treatment of ocular surface disease.

## 1. Introduction

Cornea is the transparent front part of the eye. It is composed of epithelium, Bowman's layer, stroma, Descemet's membrane, and endothelium. Limbal stem cells (LSCs) are residing at the basal layer of the limbus and could differentiate into terminal epithelium cells for replacement. In the stage of corneal damage, LSCs could generate epithelial cells for repair [1]. As damage progresses, angiogenesis and lymphangiogenesis in the avascular cornea result in the infiltration of neutrophils and macrophages as well as Th1 cells for further attack. As the pathological process involves regions of corneal limbus, LSCs are lost and dysfunctional and fail to replace the damaged epithelial cells, leading to blindness [2]. In this case, LSCs and corneal transplantation are the most feasible option to improve ocular surface damage and vision. Although the success rate of transplantation is high, graft rejection still occurs resulting from preoperative high-risk factors, postoperative inflammation, angiogenesis, lymphangiogenesis, and immune response [3–5]. To date, it has been reported that more than 10 million patients have been suffering from corneal blindness in the world [6].

Mesenchymal stem cells (MSCs) are originated from multiple adult tissues such as bone marrow, liver, and adipose tissue. As pluripotent cells, MSCs could differentiate into different cell types [7]. Besides their differentiation potential, MSCs exert immunomodulatory and anti-inflammation effects on the surrounding cells by the release of secreted cytokines [8]. When cocultured with LSCs, MSCs could stimulate LSCs proliferation and growth factor expression in vitro [9]. Therefore, MSCs therapy could be a promising approach for ocular surfaces diseases via control of lymphangiogenesis, inflammation, and immune response. In the review, we will first overview the knowledge of MSCs and then focus on how MSCs control the pathological cross talk between lymphangiogenesis and inflammation in the treatment of corneal diseases.

## 2. Characteristic and Potential of MSCs

### 2.1. Definition of MSCs

MSCs have been isolated from several adult tissues, including bone marrow, adipose tissue, liver, dental pulp, endometrium, muscle, amniotic fluid, placenta, and umbilical cord blood [10–12]. MSCs have pluripotent or multipotent properties as well as a great potential of differentiating into mesodermal cell lineages (e.g., adipocytes, osteocytes, and chondrocytes) and nonmesodermal cell lineages (e.g., cardiomyocytes, hepatocytes-like cells, neurons, astrocytes, and endothelial cells) both in vivo and in vitro. In addition, it is found that pericytes present in several organs, such as skeletal muscle and pancreas, also express the very same markers used by MSCs [13]. They could share many of the differentiation characteristics of MSCs in vitro [14]. Thus, the perivascular niche can be regarded as a subset of MSCs [13–16]. Due to the lack of specific markers for these cells, the authentic MSCs are difficult to identify. To resolve this problem, the International Society for Cellular Therapy has provided the minimum criteria for defining multipotent MSCs: plastic adherent under standard culture conditions; positive for the expression of CD105, CD73, and CD90 surface markers; absent for the expression of CD11b, CD14, CD19, CD34, CD45, CD79a, and HLA-DR surface markers; and capable of differentiating into osteocytes, adipocytes, and chondrocytes under a specific stimulus in vitro [17].

### 2.2. Differentiation Ability

MSCs have both endothelial and epithelial tissue coding genes and could be promoted to differentiate into endothelial- or epithelial-like cells both in vitro and in vivo [18–20]. Under specific conditions, MSCs could differentiate into corneal epithelial cells, keratocyte-like cells, and endothelial-like cells to repair damaged corneas [21–25]. However, some lines of evidence found that the replaced cells derived from MSCs do not behave as true tissue cells [26]. This might be due to the inconsistent differentiation protocols and heterogeneity of cell population [27, 28].

### 2.3. Immunomodulatory and Anti-Inflammation Potential

MSCs are potent regulators of immune response and inflammation. MSCs could be activated by the inflammatory microenvironment through exposure to proinflammatory cytokines, such as interferon-*γ* (IFN-*γ*), tumor necrosis factor-*α* (TNF-*α*), and effector T cells [29, 30]. In vitro, MSCs interact with innate and acquired immune response and inhibit the proliferation and function of T cells, B cells, dendritic cells, and natural killer cells [31–33]. The regulation of immune cells by MSCs mainly comes from a panel of cytokines secreted by MSCs, including IL-6, IL-10, transforming growth factor-*β* (TGF-*β*), metal matrix proteinase (MMP), prostaglandin E2 (PGE2), indoleamine-2,3-dioxygenase (IDO), human leukocyte antigen-G5 (HLA-G), and nitric oxide (NO) [34, 35]. Second, MSCs could decrease the expression levels of Th1 cell factors (IFN-*γ* and IL-2) and increase the expression levels of Th2 cell factors (IL-4 and IL-10), thereby promoting immune response of naïve CD4+ cells toward the Th2-type response [32]. Third, when cocultured with lymphocytes, MSCs produce PGE2 and TGF-*β* to promote regulatory T-cells (Tregs) differentiation and expansion [36, 37]. It is well known that Tregs have the capacity to suppress the proliferation of activated T cells. Therefore, modulation of Tregs has been suggested as the main mechanism of MSCs in maintaining immune tolerance for allografts survival in organ transplantation [38].

The application of MSCs in organ transplantation has been tested in rat and primate transplantation models. MSCs seemed to significantly suppress immune rejection and prolong graft survival in the heart, liver, kidney, pancreas, and other solid organs [39–42]. Following systemic infusion, MSCs could not only migrate to lesion but also be trapped in lungs and other organs [43]. Although it is not fully clear, MSCs homing is regulated via chemokine, chemokine receptors, intracellular signals, adhesion molecules, and proteases, such as stromal cell-derived factor-1*α* (SDF-1*α*) and C-X-C chemokine receptor type 4 (CXCR4) [44–47]. It is currently under investigation whether improved MSCs homing could bring about better therapeutic effect.

However, MSCs therapy is not always successful and even accelerates allograft rejection after organ transplantation [57]. The controversial results might be influenced by many factors including infusion time and dose, administration mode, and homing efficiency of MSCs.

### 2.4. Angiogenic Property of MSCs

A growing body of evidence has shown the regulation effect of angiogenesis by MSCs. This effect was mainly attributed to the modulation of angiogenic factors produced by MSCs. For instance, vascular epithelial growth factor (VEGF) and basic fibroblast growth factor (bFGF) both promote the migration and proliferation of vascular endothelial cells [58]. In cardiac ischemia repair, MSCs stimulate neovascularization of infarct tissue through upregulating VEGF to improve cardiac function [59]. The effects may be associated with the role of TLR2 [60]. However, the direct interaction between VEGF and TLR2 in MSCs is not clear. In addition, some reports demonstrated that MSCs could be additional important cells for proangiogenesis to form provisional granulation matrix in the proliferation phase of wound healing [61].

Different from the effect of proneovascularization in ischemic tissues and tumors, MSCs showed an opposite effect on inflammation-related corneal angiogenesis after chemical injury. This action was associated with the significantly smaller mean neovascularized area and a reduced expression level of VEGF [62], which might be attributed to the expression of high level of thrombospondin-1 (TSP-1), which inhibits VEGF [63]. These results suggested that the different microenvironment would modulate different behavior and function of MSCs. Below, we overviewed MSCs applications in ocular surface diseases.

## 3. MSCs in Ocular Surface Diseases (Table 1)

### 3.1. Corneal Wound Repair

Corneal injury is caused by thermal injury, alkali or acid burns, and immune or hereditary disorders and leads to corneal inflammation, neovascularization, conjunctivalization, impaired vision, and even blindness [64]. LSC is an essential cell population for corneal epithelium regeneration and ocular surface reconstruction. Unfortunately, LSC loss and dysfunction in corneal limbus trigger severe inflammation and neovascularization [65]. To be noted, LSC transplantation remains as the effective strategy to treat LSCD but it is challenged by limited donors and allograft rejection [66, 67].

Alternative therapy is to improve resident LSC and corneal epithelial cell (CEP) expansion for repair [10, 68, 69]. In vitro studies showed that MSCs could stimulate LSC and CEP proliferation when they were cocultured. Both systemic and topical administration of MSCs have been shown to accelerate corneal regeneration and healing [48, 49]. In practice, to increase local concentration, MSCs were injected into the injured cornea with a hollow plastic tube [70], through subconjunctival administration of MSCs [62] or through transplantation of MSCs with tissue engineering materials, such as amniotic membrane (AM) [50, 71] and nanofiber scaffolds [72]. The strategy of combining with tissue engineering materials is better for cornea recovery than MSCs or tissue engineering materials used alone [51].

Besides stimulation of LSC proliferation, MSCs injection effectively alleviates inflammation and neovascularization in the injured cornea. MSCs increased the expression of the anti-inflammatory cytokines IL-10, TGF-1, TNF-*α*-stimulated gene/protein 6 (TSG-6), and the antiangiogenic factor thrombospondin-1 (TSP-1) and reduced the expression levels of the proinflammatory factors IL-2, IFN-*γ*, IL-17, macrophage inflammatory protein-1*α* (MIP-1*α*), and MMP-2 [70, 71, 73, 74]. Ultimately, neutrophil and macrophage infiltration is largely reduced.

### 3.2. Corneal Transplantation

Corneal transplantation is the most common form of human tissue transplantation. Comparing with other types of organ transplantation, normal-risk corneal transplants have an exceptionally high success rate of up to 80% over 5 years, which is mainly based on the specific immune privilege of cornea including low-level expression of MHC I and MHC II, the lack of indigenous antigen-presenting cells (macrophages or Langerhans cells), the absence of lymphoid and blood vessels, and anterior chamber associated immune deviation (ACAID) [75–77]. However, as risk factors, preexisting lymphangiogenesis and blood vessel together with inflammation promote graft injection [78].

Currently, a broad range of treatment strategies have been proposed to increase the duration of grafts survival. The leading method for preventing transplant rejection is corticosteroids and immunosuppression such as cyclosporine A, FK-506 [79–81]. However, long-term immunosuppression could produce drug toxicity and potential complications; the used dosages are limited. More recent works have focused on endothelia transplantation (only endothelia are diseased) and lamellar transplantation (endothelia are not diseased) to reduce immune rejection [82–84].

MSCs administration has been widely tested in corneal transplantation and inconsistent results have been reported. Some studies demonstrated that pretransplant infusion of MSCs was effective to prolong graft survival; meanwhile, MSCs used postoperatively are less effective, especially for kidney and heart transplantation. This might be explained by the fact that preoperative infusion of MSCs modulated Tregs expansion and induced immune tolerance before occurrence of inflammation and immune progress [85–87]. Oh et al. [52] suggested that pretransplant systemic infusion of human MSCs inhibited immune response largely due to suppressing early inflammation caused by surgery and reducing the activation of antigen-presenting cells (APCs) in both cornea and draining lymph nodes (DLNs). The role of MSCs was primarily exerted by secreting the soluble anti-inflammatory protein TSG-6. However, Jia et al. [53] have shown that MSCs prolonged corneal allograft survival time only when injected immediately after the surgery and preoperative administration exerted no significant effect. The cornea immune privilege triggers delayed-type hypersensitivity which may take longer for the corneal allograft to activate an immune response than other types of solid organ transplantation.

By contrast, Oh et al. [88] were the first who examined the immunomodulatory effects of MSCs in corneal transplantation in a pig-to-rat model. Allogeneic rat MSCs were applied topically to corneal grafts for 2 h immediately after transplantation. They observed the increased expression levels of Th2-type cytokine (IL-10) in the rejected grafts from MSC-treated rats and a shift from Th1 to Th2 cell type following MSCs administration. However, MSCs injection failed to prolong pig corneal xenograft survival in rats. Similarly, Fuentes-Julián et al. [89] obtained an insufficient conclusion about adipose-MSCs treatment to prevent corneal grafts rejection. In the study, local or systemic adipose-MSCs administration in rabbit corneal transplantation models at normal- or high-risk rejection does not prolong the graft survival. The used adipose-MSCs lacked immunomodulatory ability on T lymphocytes and immunophenotypical secretion molecules, which may be the reason why the adipose-MSCs destroyed the innate ocular immune privilege and accelerated rejection [89].

All of the above observations demonstrated that MSCs would be a potential therapeutic tool for corneal allograft transplantation and the molecular mechanisms of action need to be further studied. Importantly, many factors including the source of MSCs, the infusion time, the dose of injection, and the mode of administration would influence the best results of MSCs treatment on corneal transplantation. In addition, MSCs in combination with immunomodulatory drugs are an alternative treatment. Intravenous transfusion of MSCs and cyclosporine A (CsA) achieved a synergistic effect on suppressing immune rejection of corneal grafts [53].

### 3.3. Dry Eye Syndrome

Dry eye syndrome (DES) or keratoconjunctivitis sicca (KCS) is the major ocular surface disease affecting ranges from 7% to 33% of the worldwide population [90, 91]. Dry eye is a common ocular complication associated with chronic graft-versus-host disease (GVHD) after allogeneic hematopoietic stem cell transplantation, occurring in 60% of patients [92, 93]. DES is characterized by deficiency of the tear film components (lipid, aqueous, and mucin). Although the mechanisms of DES are yet unknown, inflammation in the lacrimal gland and the ocular surface plays a key role in the pathogenesis of the disorder. DES is also an autoimmune disease with immune-mediated destruction in the whole process [94, 95]. Immunohistochemistry of lacrimal gland shows immune cell infiltration and loss of acinar epithelial cells, and the expression of proinflammatory cytokines is increased [96]. Studies have suggested that the proinflammatory factors inhibit neurotransmitter release resulting in insufficient secretion of lacrimal gland [97]. In addition, the lacrimal gland could be involved as a target in several systemic and autoimmune diseases including Sjögren syndrome, sarcoidosis, and chronic GVHD [96]. The current treatment strategies, including tear replacement, anti-inflammatory drugs, and punctual occlusion, often fail to resolve the underlying problems of DES.

Topical application of MSCs could be a safe and available treatment for periocular diseases with immune involvement, such as KCS/DES [98, 99]. Allogeneic adipose-MSCs were implanted around lacrimal glands in dogs with KCS [100]. The implanted cells effectively reduced clinical signs during a 9-month follow-up. Lee et al. [55] also demonstrated that periorbital administration of MSCs could protect the ocular surface in a murine model of DES. In the intraorbital gland and ocular surface, the CD4+ T-cells infiltration was reduced. MSCs suppressed inflammation and increased aqueous tear production [55]. In a recent clinical practice, 22 patients with refractory dry eye secondary to chronic GVHD were treated with MSCs, 12 of whom showed reduced symptoms with improved dry eye scores. The results were accompanied by increasing the number of CD8+CD28−T cells, which suggest that MSCs regulate the balance between Th1 and Th2 [56]. There is a human Phase I/II clinical trial involving allogeneic MSCs treatment for primary Sjögren syndrome (http://clinicaltrials.gov/ct2/show/NCT00953485). Based on the double effect between inflammation and immunomodulation, MSCs are a promising source to treat dye eye syndrome.

## 4. Summary and Perspectives

**Figure 1:**
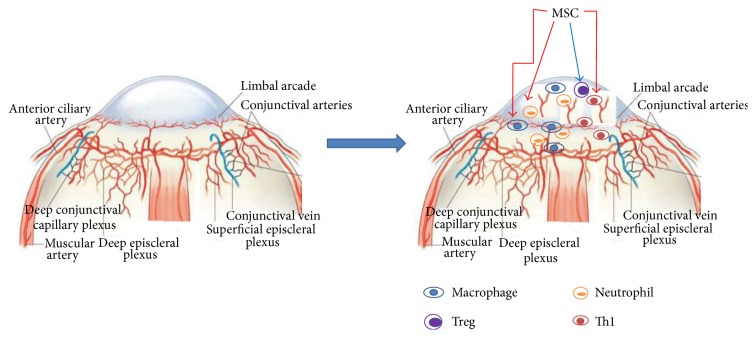
Modulation of immune cells and inflammatory cells by MSCs in corneal surface diseases. Cornea is the avascular and transparent front part of the eye, maintained by immune and angiogenesis privilege. In the occurrence of injury and transplantation, ingrowth of blood and lymph vessels into the cornea leads to infiltration of inflammatory cells and Th1 cells, which strengthen the inflammation and damage the cornea structure. MSCs have several protective functions by (1) inhibition of the inflammatory cell infiltration and inflammatory cytokine release, (2) activation of Treg cells for immune tolerance, (3) tuning the transition from Th1 cells toward Th2 cells, and (4) improving epithelium regeneration (not shown).

The ocular immune and angiogenic privileges act as a barrier to protect corneal function. Ingrowth of new blood vessels orchestrates inflammatory cell infiltration leading to inflammation and impaired epithelial cell repair. MSCs have shown therapeutic effect in corneal surface diseases by several lines of mechanisms: inhibition of inflammatory cell infiltration and inflammatory cytokine release, modulation of the switch from Th1 cell type toward Th2 cell type, activation of Treg cells, and stimulation of epithelial cell regeneration (Figure 1). Therefore, MSCs would be a very promising tool in the treatment of corneal diseases. Further studies would focus on increase of MSCs homing efficiency, time, and safety and MSCs administration, together with evaluation of MSC-based therapies when dealing with ocular surface diseases.

## Figures and Tables

**Table 1:** Experiments of MSCs in ocular surface diseases.

Diseases	Experimental outcomes	The mechanisms	Factors	References
Chemical injury	Reduce corneal opacity	Reduce inflammation and neovascularization	↑ TSG-6	[48]

Chemical injury	Protect the corneal surface	Reduce inflammation and neovascularization Reduce CD4+ cells infiltration	↑ IL-10, IL-6, TSP-1, and TGF-*β*1 ↓ IL-2, IFN-*γ*, and MMP-2	[63]

Alkali burn	Improve wound healing	Enhance the recovery of corneal epithelium Decrease the CNV area	↓ MIP-1*α*, TNF-*α*, and VEGF	[62]

Chemical burn	Restructure damaged corneal surface	Inhibit inflammation and angiogenesis	↓ IL-2 and MMP-2	[71]

Chemical burn	Affect profiling of IL-17-secreting cells	Mainly modulate non-Th17 cells and partially suppress Th17 cells	↓ IL-17	[73]

Corneal allotransplantation	Prolong grafts survival	Inhibit immune response Suppress early inflammation Reduce the activation of APCs	↑ TSG-6	[52]

Corneal allotransplantation	Prolong grafts survival	Prevent T-cells response Regulate the balance of Th1/Th2 to Th2 Increase CD4+CD25+Foxp3+ Treg	↑ IL-10 and IL-4 ↓ IL-2 and IFN-*γ*	[53]

Corneal allotransplantation	Prolong grafts survival	Reduce NK cells infiltration Increase CD4+ Foxp3+ Treg Suppress peripheral immune response Promote an immunoregulatory milieu	↓ IL-6, IL-1*β*, and IFN-*γ*	[54]

DES	Protect ocular surface	Reduce the CD4+ T cells		[55]

Dry eye secondary to chronic GVHD	Reduce clinical symptoms and improve dry eye scores	Increase the CD8+CD28− T cells Regulate the balance of Th1/Th2 to Th2		[56]

TSG-6: TNF-*α*-stimulated gene/protein 6; MMP: metal matrix proteinase; CNV: cornea new vessel; TSP-1: thrombospondin-1; TNF-*α*: tumor necrosis factor-*α*; MIP-1*α*: macrophage inflammatory protein-1*α*; IFN-*γ*: interferon-*γ*; VEGF: vascular epithelial growth factor; DES: dry eye syndrome; GVHD: graft-versus-host disease; APCs: antigen-presenting cells.
